# Editorial: Embodied Cognition Over the Lifespan: Theoretical Issues and Implications for Applied Settings

**DOI:** 10.3389/fpsyg.2018.00550

**Published:** 2018-04-20

**Authors:** Annalisa Setti, Anna M. Borghi

**Affiliations:** ^1^School of Applied Psychology, University College Cork, Cork, Ireland; ^2^Department of Dynamic and Clinical Psychology, Sapienza Università di Roma, Rome, Italy

**Keywords:** emobodied cognition, aging and cognitive function, cognitive development, lifespan, virtual reality, action, abstract

## Introduction

The Special Topic on Embodied Cognition over the Lifespan and in Applied Settings aimed at gathering evidence on the role of EC in development, adulthood, and aging, and to shed light on the applied fields benefiting from this approach. The collection originating from it shows the variety and richness of studies falling under this umbrella. The collection is organized in five sections: the first outlines new perspectives in the potential applications of EC to cognitive development and healthy aging; the second, third, and fourth are dedicated to studies on childhood, older age, and young adults. Each section comprises work on action and language; the fifth section is dedicated to instruments and techniques to utilize within an EC framework, such as Virtual Reality (VR).

## Perspectives

This section collects proposals of new perspectives and directions of research on embodiment over the lifespan and their implications for social practices. Glenberg and Hayes propose a strongly embodied explanation of two phenomena characterizing childhood—infantile amnesia, consisting in the scarcity of autobiographical memories from early childhood, and childhood amnesia, the quick forgetting of situations until age of seven—and extend the same explanation to cognitive decline with aging. They ascribe a crucial role to the development of the hippocampus, the primary function of which is that of spatial navigation. Before locomotion, they propose that infants have difficulties in associating events with locations, because places are not strongly distinguished by spatial codes, and in memorizing similar events experienced in different places. When locomotion starts, place and grid cells of the hippocampus adapt to the environment and places become stable memory cues. The maturation of locomotion abilities further facilitates long-term episodic memory and lead to the end of infantile amnesia and of childhood amnesia. Within the same framework, the reduction in self-locomotion abilities is at least partially responsible of the cognitive decline with aging.

Costello and Bloesch propose that Embodied Cognition can enhance our understanding of the changes occurring in sensory processing, mental representations, and action perception with aging. They suggest that older adults may rely more on visual inputs than younger adults, possibly due to the degradation of their internal action models, therefore potentially becoming more disembodied. EC can also help understanding the diversity of the aging process across individuals, whereby physical and cognitive aging, particularly perceptual and motor skills interplay with cognitive abilities. Importantly, the role the environment emerges as facilitator or barrier to healthy aging.

Hommel and Kibele outline the principles of an embodied-cognition approach to aging, according to which abilities are not a static endowment but rather emerge from fruitful and active exchanges with the environment. The progressive reduction of resources could thus be intended as a modification in the relationship with the environment, that requires reframing. They contend that healthy aging relies on the systematic use of control resources; these resources, even if present, are often not fully exploited. They discuss three phenomena characterizing aging, i.e., the reduction of dopaminergic supply, loneliness, and the loss of body strength, that might lead to the loss and lack of maintenance of cognitive control, leading to a vicious spiral of demotivation and further lack of strategies to contrast cognitive decline. While further research on how use of cognitive control resources affects their maintenance, social practices should promote healthy aging supporting self-empowerment strategies of individuals and reconsidering current policies as retirements laws.

## Children

### EC and language in children

In this section, contributions investigate how language acquisition is influenced by embodied and grounded aspects. Barca et al. show that the extensive use of the pacifier, i.e., of a device actively involving the mouth motor system, influences the acquisition of abstract and emotional concepts, leading to a different pattern of conceptual relations in children who have used it (beyond age 3). The authors interpret their work in the framework of the embodied Words As social Tools (WAT) view (Borghi et al., 2017), according to which abstract concepts activate the linguistic system and the social dimension more than concrete concepts.

Thill and Twomey explore with a computational study the factors affecting Age of Acquisition (AoA) of words; they show that factors traditionally considered in literature such as concreteness, imageability and frequency do not account for the variability of AoA data. AoA can be explained, however, when recurring to “groundability” in sensorimotor and interoceptive experience, i.e., considering concepts as richly embodied.

In the same vein, Inkster et al. study the influence of words' rated body–object interaction (BOI) and imageability in 6–7 years-old and in adults. For the first time they obtain BOI ratings from parents of children (child-BOI), and compare the influence on auditory naming task of high and low imageability words and of high and low child-BOI words. Results showed that children's auditory naming times are influenced by both imageability and child-BOI, testifying the influence of embodiment and groundedness on conceptual representation even in very young children.

Adams provides substantial evidence from the literature that learning a second language should also be grounded in action. In bilingual individuals, both the first and the second language display compatibility effects between semantic content and action. Importantly, children with Specific Language Impairment are known to present motor deficits, therefore they can potentially benefit from the involvement of the body in language learning.

### Children, body, sensori-motor information

In this section the majority of contributions focuses on the role played by action and movement for cognitive activity in children.

Lozada and Carro used a classical task, the Piagetian conservation task, in 6–7 years-old. They clearly demonstrated the effects of action on cognition: actively manipulating the materials led children to comprehend conservation phenomena better than when they merely observed an adult's demonstration.

In a different perspective, Wunsch et al. tested children aged 3–10 and adults in three motor tasks (e.g., the bar-transport task) and three cognitive tasks on executive functions (e.g., the Tower-of-Hanoi task). They found a general improvement of the performance with age, but also found that, when age was controlled, there was no relationship between motor and cognitive tasks. These results cast doubts on the assumption of a strict interrelationship between action and cognition proposed by embodied and grounded cognition approaches.

Whether the human body and actions can be utilized to support cognitive tasks has also been explored by Pouw et al. The authors looked at the role of utilizing body analogy in explaining physical systems to school age children. They found that instructional animations based on human body were particularly useful for children with lower maths skills for a specific task, while transfer of learning did not benefit from it.

One final study on children focuses on the development of the ability to respond to stimuli in the environment with the appropriate grasp. Richez et al. ask 8–11 years old children to respond to the color of a stimulus presented at different distances from the body. Only over 10 years of age, children responded faster in grasping a proximal switch when they were shown a proximal stimulus, and a distal switch when they were presented with a distal stimulus. The authors argue that this is in line with the view according to which, between 8 and 10 years, children prepare their body to more efficiently act in the environment.

Ninaus et al. investigated the Spatial-Numerical Associations of Response Codes across the lifespan, showing that it increases with age, reflecting the increased experience with the association between numbers magnitude and spatial physical space, which is culturally determined. On the contrary, the interference of flanking numbers on a line bisection task forms a U shape across the lifespan, suggesting that it is grounded in inhibition abilities, which are weaker in children and older adults.

These mixed results, with some positive and some negative findings, highlight that the body plays a complex role in cognitive development, that varies depending on the age, the kind of task and the complexity of the required cognitive skills.

### The role of action and technology in children's development

Hainselin et al. test the enactment effect (EE), consisting in better memory for performed actions than for verbally encoded action sentences, in 6–10 years-old children. Overall, action conditions (direct action and action observation) were better than non-action conditions (verbal and listening tasks). Even if younger children memory performance was worse than that of older children, this difference disappeared during learning through action. Their results suggest that episodic memory might not underlie EE in young children, and highlights the importance of action for learning and memory.

Galetzka offers a commentary of a paper of Radesky et al. (2015) according to which technological advances are so fast that research on their impact on children's cognitive processes cannot keep up with them. The author reviews controversial results of research on the effect of interactive technologies as tablets and smartphones. He points out that, while initial results suggested that interacting with tablets was an impoverished experience compared to interacting with objects, when children actively interact with them through touchscreens the typical transfer difficulties characterizing traditional media as television is absent. Hence the advantages of real-life over technology-based learning are highly context-dependent.

Miklashevsky and Fischer adopt the approach outlined by Hommel and Kibele, according to which cognition changes in the dynamical interchange with the environment, to investigate how development changes in the light of the pervasive digitalization and of the extensive use of smartphones and tablets in children. They argue that using oral instead of typed language will likely link linguistic representation to the orofacial rather than to the manual activity, the reduction of hand motor activity in interacting with objects will likely lead to changes in spatial representation and in abstract conceptualization, for example replacing manual with visual metaphors (see the point vs., grasp the idea), and the reduction of finger counting will render individual numbers less accessible; finally, the absence of direct social interaction will reduce the pragmatic value of language, rendering it less linked to action.

## Aging

When looking at cognitive development the importance of an embodied perspective emerges clearly; however very few studies have attempted to implement it in understanding age-related changes on the other side of the lifespan, in older people.

### Language, memory, executive functions

Borghi and Setti focus on how older people represent abstract concepts and words. They propose an account of conceptual decline in elderly derived from the embodied WAT view (Borghi et al., 2017). The WAT view predicts that in older the decline of abstract concepts should be less marked than that of concrete ones, because the first rely more heavily on language. This contrasts with the view according to which abstract concepts should decline more, because abstract words are typically acquired earlier (AoA) and less frequent than abstract ones. The authors critically analyze current evidence, showing that either the well know advantage in processing of concrete over abstract words (concreteness effect), is reduced with age, or that linguistic-based compensatory strategies are activated. This evidence gives preliminary support to the WAT view.

Vallet et al. start from an embodied view, according to which episodic and semantic memory are grounded in sensorimotor system. They develop and illustrate a memory test, the SEMEP (semantic-episodic) memory test. Results of the test show very distinct pattern of performance across older groups. All older individuals, with or without cognitive deficits, committed more confusion errors than young adults, likely due to a degradation in perception with age. Alzheimer patients showed severe impairments in episodic memory and committed more intrusion errors than the other groups, likely due to difficulties in multimodal integration; finally, semantic dementia patients showed the most severe deficits of semantic memory.

Temporal information processing, the temporal accuracy with which we perceive external stimuli has been linked with efficient cognitive performance; Nowak et al. show the link between perception, assessed by temporal information processing, and executive functions in older adults. They find a correlation between temporal information processing and performance in the Tower of Hanoi.

### Action, perception and cognition

As highlighted by both Hommel and Kibele and Costello and Bloesch it is important to understand the role of embodiment and action in aging, potentially shedding a new light on cognitive deficits and cognitive resources in aging.

Creativity is a well-researched topic in young adults, however much less research is devoted to creativity in older. Kuo and Yeh demonstrate that walking enhances divergent thinking. Specifically, older adults can reach the same performance level in a divergent thinking task, when they are free to choose their own walking path as opposed to walking on a fixed path.

Niu et al. in an ERP study, show that a general decline on action preparation occurs with aging, and an age-related impairment on the effect-action retrieval of intention-based actions, possibly due to decline in associative memory.

Kim et al. look at bimanual instrument playing (electronic drum), requiring coordination, in young adults, as well as healthy and Mild Cognitive Impairment older adults. They show that the ability to synchronize with an external cue correlates with cognitive skills; this indicates the close link between action and cognition in aging.

## Embodied cognition in young adults

### Language and embodiment

According to the conceptual metaphor theory, mappings are formed between abstract and concrete concepts. Chen et al. test the hypothesis that abstract-abstract mappings can be formed focusing on the concept of time and abstract action. Results of two lexical decision tasks revealed robust priming effects when a target verb and its prime (length-changing line or sound beep) lasted the same time (time consistent condition). The authors propose that mappings between concepts are influenced by common elements, either linguistic or embodied, that are differently recruited depending on the conceptual difficulty.

Chen et al. look at the role of emotions in words processing. In addition to replicating the effect of priming for words of the same emotional valence, they show that words referring to life-events also activate specific emotions, however it happens at a later stage, as shown by the longer Stimulus Onset Asynchrony compared with priming obtained when valence, as opposed to specific emotions, is manipulated.

### Action, body, motor resonance

Capellini et al. investigate an exquisitely embodied phenomenon, that of motor resonance, i.e., the activation of a simulation while observing the actions of others. Italian participants were presented with videos of an Italian (in-group) or Arabian (outgroup) individuals with a neutral (box of juice) vs. a threatening object (gun) and measured mouse tracker responses. When a threatening object was present, responses were delayed with outgroup members, revealing a higher level of motor resonance with the in-group and the activation of threat-related stereotypes induced by the context.

Xie et al. investigate a debated topic in the embodied literature: metaphorical associations. In two experiments, they manipulated the kind of seat where participants were sitting and recorded performance in a memory task and in a creativity task. The soft seat was associated with better performance in the creativity task, while the hard seat was associated with better performance in the memory task.

## Instruments

Repetto et al. propose to use VR to improve memory function in elderly. Glenberg and Hayes highlighted the link between locomotion and episodic memory, by means of the alignment of place cells, encoding location, and grid cells updating the viewpoint when self-motion occurs. VR allows the individual to experience a sense of moving in the environment with much limited real movement. Several studies show that episodic memory can be enhanced by using VR exploration, therefore, the authors propose that VR should be exploited to support episodic memory in older adults, who have often reduced mobility.

Johnson-Glenberg et al. are also interested in embodiment through technology. Specifically, they assess the efficacy of different levels of immersiveness and embodiment in supporting learning in college students. They assess both declarative knowledge and generative knowledge immediately after the lesson and at 1 week follow up and found that there was a significant difference after a week, with the more embodied class benefitting more the generative knowledge tests.

## Conclusion

The articles collected in this Special Topic trace novel avenues to study Embodied Cognition and its contribution to identifying strengths and weaknesses across the lifespan, and techniques to support the former and prevent the latter.

## Author contributions

All authors listed have made a substantial, direct and intellectual contribution to the work, and approved it for publication.

### Conflict of interest statement

The authors declare that the research was conducted in the absence of any commercial or financial relationships that could be construed as a potential conflict of interest.
